# Muramyl dipeptide potentiates *Staphylococcus aureus* lipoteichoic acid-induced nitric oxide production via TLR2/NOD2/PAFR signaling pathways

**DOI:** 10.3389/fimmu.2024.1451315

**Published:** 2024-12-06

**Authors:** Jintaek Im, Jun Ho Jeon, Dongwook Lee, Jeong Woo Park, Woohyung Jun, Suwon Lim, Ok-Jin Park, Cheol-Heui Yun, Seung Hyun Han

**Affiliations:** ^1^ Department of Oral Microbiology and Immunology, and Dental Research Institute, School of Dentistry, Seoul National University, Seoul, Republic of Korea; ^2^ Department of Agricultural Biotechnology, and Research Institute of Agriculture and Life Sciences, Seoul National University, Seoul, Republic of Korea; ^3^ Institutes of Green-bio Science and Technology, Seoul National University, Pyeongchang, Republic of Korea

**Keywords:** *Staphylococcus aureus*, nitric oxide, lipoteichoic acid, muramyl dipeptide, peptidoglycan, macrophages

## Abstract

Lipoteichoic acid (LTA) and peptidoglycan (PGN) are considered as key virulence factors of *Staphylococcus aureus*, which is a representative sepsis-causing Gram-positive pathogen. However, cooperative effect of *S. aureus* LTA and PGN on nitric oxide (NO) production is still unclear despite the pivotal roles of NO in initiation and progression of sepsis. We here evaluated the cooperative effects of *S. aureus* LTA (SaLTA) and muramyl dipeptide (MDP), the minimal structure of PGN, on NO production in both a mouse macrophage-like cell line, RAW 264.7 and mouse bone marrow-derived macrophages (BMMs). Although MDP alone did not affect NO production, MDP potently enhanced SaLTA-induced NO production via the expression of inducible NO synthases. The enhanced NO production was ameliorated in BMMs from TLR2-, CD14-, MyD88-, and NOD2-deficient mice. Moreover, the augmented SaLTA-induced NO production by MDP was attenuated by inhibitors specific for PAFR and MAP kinases. Furthermore, MDP also potently increased SaLTA-induced activities of STAT1, NF-κB, and AP-1 transcription factors, and specific inhibitors for these transcription factors suppressed the elevated NO production. Collectively, these results demonstrated that MDP potentiates SaLTA-induced NO production via TLR2/NOD2/PAFR, MAP kinases signaling axis, resulting in the activation of NF-κB, AP-1 and STAT1 transcription factors.

## Introduction

Sepsis, a life-threatening medical complication caused by microbial infection, comprises a systemic inflammatory response syndrome followed by critical physiological changes, including systemic hypotension, vasodilation, hypothermia, and multi-organ dysfunction (1). In fact, approximately 30 million cases of sepsis were annually reported worldwide, together with a high mortality rate ranging from 16% to 33% (2). Although sepsis is caused by various microbial infections, such as bacteria, viruses, and fungi, bacterial infection is the most common. Case of sepsis by Gram-positive bacterial infection has been on the rise due to the increasing proportion of hospital-acquired infection, where its frequency is now comparable to that by Gram-negative bacterial infection (3). In bacterial culture of blood from sepsis patients, Gram-negative and Gram-positive bacteria were detected from 42% and 33% of patients, respectively (4).

Nitric oxide (NO) is an amphiphilic radical gas that is produced by the activation of three different NO synthases (NOS) (5). The endothelial NOS (eNOS) and neuronal NOS (nNOS) produce nanomolar levels of NO and are constitutively expressed in epithelial and neuronal cells, respectively. In contrast, inducible NOS (iNOS), mainly expressed in immune cells, including macrophages (6), induces micromolar concentrations of NO in response to various stimuli, including pathogen-associated molecular patterns (PAMPs), and proinflammatory cytokines, such as tumor necrosis factor-α (7). Although it is involved in various biological processes, such as host defense during microbial infection, immune regulation, and blood pressure control, excessive NO production, especially by iNOS, is related to pathogenicity of sepsis (8). According to the previous studies, overproduction of NO resulted from excessive iNOS activation aggravated systemic hypotension, hypothermia, vasodilation, and multi-organ dysfunction in sepsis (9). In fact, iNOS-deficient mice showed a decreased mortality as well as mitigated sepsis symptoms in cecal ligation-induced sepsis model (10). Furthermore, iNOS inhibitor, such as 1400W, alleviated the sepsis-induced mortality and symptoms of sepsis (11). Therefore, NO is considered as one of the key molecules responsible for the progression of sepsis.

*Staphylococcus aureus* is considered as a representative Gram-positive pathogen causing numerous human diseases, including pneumonia and endocarditis (12). Moreover, *S. aureus* is a major sepsis-causing etiologic agent, which is one of the most frequently isolated pathogens from patients with sepsis among Gram-positive bacterial infections (3). However, since underlying mechanisms responsible for *S. aureus*-induced sepsis are not fully understood, any effective treatment or vaccine against the infection is not yet developed.

Among the numerous components of *S. aureus*, lipoteichoic acid (LTA) and peptidoglycan (PGN) are considered as major cell wall constituents responsible for virulence of *S. aureus* (13). LTA, commonly found in Gram-positive bacteria, is involved in various biological processes of bacteria, including biofilm formation and adherence to the host (14). LTA can induce various inflammatory mediators in the host via Toll-like receptor (TLR) 2-mediated signaling pathway (15). On the other hand, although PGNs are found in both Gram-negative and Gram-positive bacteria, these are generally recognized by nucleotide-binding oligomerization domain (NOD) 1 and NOD2, respectively (16) that triggers signaling cascade to produce various inflammatory cytokines, chemokines, and lipid metabolites (16).

Associated with pathogenicity of sepsis caused by *S. aureus* infection, the previous studies reported that LTA together with PGN from *S. aureus* (SaLTA), but not SaLTA or PGN alone, induces severe inflammation resulting in sepsis and multi-organ dysfunction (13, 17). It is important to note that cooperatively enhanced NO production by SaLTA together with PGN in macrophages was a key causation responsible for sepsis. However, these cooperative effects of SaLTA and PGN on induction of sepsis and NO production are still controversial, which might be due to structural damage and contamination issues of LTA and PGN during their purification process (18, 19). For the reason, to gain insight for the pathogenicity of *S. aureus*-induced sepsis, we evaluated the cooperative effect of structurally-intact and highly-pure SaLTA and synthetic muramyl dipeptide (MDP), the minimal structure of PGN, on the NO production from macrophage by examining its intracellular signaling cascade.

## Materials and methods

### Reagent and chemicals

MDP and *Escherichia coli* lipopolysaccharide (LPS) were obtained from InvivoGen (San Diego, CA, USA). Nifuroxazide, BAY11-7082, and T5224 were purchased from Sigma-Aldrich (St. Louis, MO, USA), and CV6209, SB203580, U0126, and SP600125 were purchased from Calbiochem (Darmstadt, Germany). All broths for bacteria culture were obtained from BD Biosciences (San Diego, CA, USA). Antibodies for Western blot analysis of non-phosphorylated and phosphorylated signal transducer and activator of transcription 1 (STAT1, Catalog No. 9172 and 7649) and STAT3 (Catalog No. 9139 and 9131) were obtained from Cell Signaling (Beverly, MA, USA). Antibodies for Western blot analysis of iNOS (Catalog No. 06-573) and β-actin (Catalog No. SC-47778) were obtained from Upstate (Lake Placid, NY, USA) and Santa Cruz Biotechnology (Santa Cruz, CA, USA), respectively. For flow cytometric analysis, antibodies specific to TLR2 conjugated with phycoerythrin (PE, Catalog No. 12-9021-82), and to CD14 conjugated with fluorescein isothiocyanate (FITC, Catalog No. 123308) were obtained from eBioscience (San Diego, CA, USA) and Biolegend (San Diego, CA, USA), respectively, while antibody specific to NOD2 conjugated with PE (Catalog No. NB100-524PE) was purchased from Novus (Los Angeles, CA, USA).

### Bacteria culture and LTA preparation

To acquire pure and structurally-intact LTAs, four species of bacteria, including *S. aureus*, *Streptococcus mutans*, *Lactobacillus plantarum* and *Bacillus cereus*, were cultured based on their optimal growth culture condition (**Table 1**) and their LTAs were purified by a series of butanol extraction, hydrophobic-interaction column chromatography, and ion-exchange column chromatography as previously described (20).

**Table 1:** Information of bacteria used in LTA purification.

Bacteria species and strain	Source^a^	Culture condition
*Staphylococcus aureus* ATCC 6538	ATCC	Aerobic and shaking culture in Tryptic soy broth
*Streptococcus mutans* ATCC 25175	ATCC	Aerobic and static culture in Todd-Hewitt broth
*Lactobacillus plantarum* KCTC 10887BP	KCTC	Aerobic and static culture in De Man, Rogosa and Sharpe broth
*Bacillus cereus* KCTC 13153	KCTC	Aerobic and shaking culture in Tryptic soy broth

a ATCC, American Type Culture Collection; KCTC, Korean Collection for Type Cultures.

### Preparation of bone marrow-derived macrophages and cell culture

Wild-type C57BL/6 mice were obtained from OrientBio (Gyeonggi-do, Republic of Korea). TLR2- or MyD88-deficient C57BL/6 mice were kindly provided by Prof. Shizuo Akira at Osaka University (Osaka, Japan), while CD14- and NOD2-deficient mice were obtained from Jackson Laboratory (Bar Harbor, ME, USA). All animal experiments were approved by the Institutional Animal Care and Use Committee of Seoul National University (Approval Number: SNU-210403-1-2). To generate bone marrow-derived macrophages (BMMs), bone marrow cells were isolated from the femurs and tibiae of mice, and differentiated for 6 days in Dulbecco’s modified Eagle’s medium (DMEM; HyClone, Logan, UT, USA) containing 10% fetal bovine serum (FBS; HyClone), and M-CSF at 20 ng/ml (PeproTech, Rocky Hill, NJ, USA) at 37°C (21). RAW 264.7, a mouse macrophage-like cell line, and BMMs from TLR2-, MyD88-, CD14- or NOD2-deficient mice were cultured in DMEM containing 10% FBS at 37°C.

### NO production

Nitrite was examined as an indication of NO level in the culture supernatant (22). RAW 264.7 cells or BMMs (200 μl of 5 × 10^5^ cells/ml) were treated with LTA (0, 1 or 3 μg/ml) and/or MDP (0, 3 or 10 μg/ml) for 24 h. For experiments using inhibitors for MAP kinase or transcription factors, the cells were pre-treated with each inhibitor for 1 h before the stimulation with SaLTA (0 or 3 μg/ml) and/or MDP (0 or 10 μg/ml) for an additional 24 h. The cell culture supernatants were then mixed with Griess reagent (2% phosphoric acid, 1% sulfanilamide, and 0.1% naphthylethylenediamine dihydrochloride). NaNO_2_ was used as a standard. The absorbance was measured at 540 nm using a microplate reader (Molecular Devices, Sunnyvale, CA, *USA*).

### Real-time quantitative polymerase chain reaction

The mRNA expression levels of iNOS, and interferon (IFN)-β, were evaluated by RT-qPCR. Briefly, RAW 264.7 cells (3 ml of 5 × 10^5^ cells/ml) were treated with SaLTA (0, 1 or 3 μg/ml) and/or MDP (0, 3 or 10 μg/ml) for 12 h. After purifying total RNA using TRIzol reagent (Invitrogen, Carlsbad, CA, USA), complementary DNA (cDNA) was synthesized using one microgram of total RNA and reverse transcriptase (Promega Corporation, Madison, WI, USA). The cDNA was subjected to StepOnePlus real-time system (Applied Biosystems, Waltham, MA, USA) with primers specific for iNOS (forward: 5’-CGCTTGGGTCTTGTTCACTC-3’, reverse: 5’-GGTCATCTTGTATTG TTGGGC TG-3’), IFN-β (forward: 5’-GCCTTTGCCATCCAAGAGATGC-3’, reverse: 5’-ACACTGTCTGCTGGTGGAGTTC-3’) and GAPDH (forward: 5’-CATCACTGCC ACCCAGAAGACTG-3’, reverse: 5’-ATGCCAGTGAGCTTCCCGTTCAG-3’).

### Immunofluorescence staining

**Figure 1:**
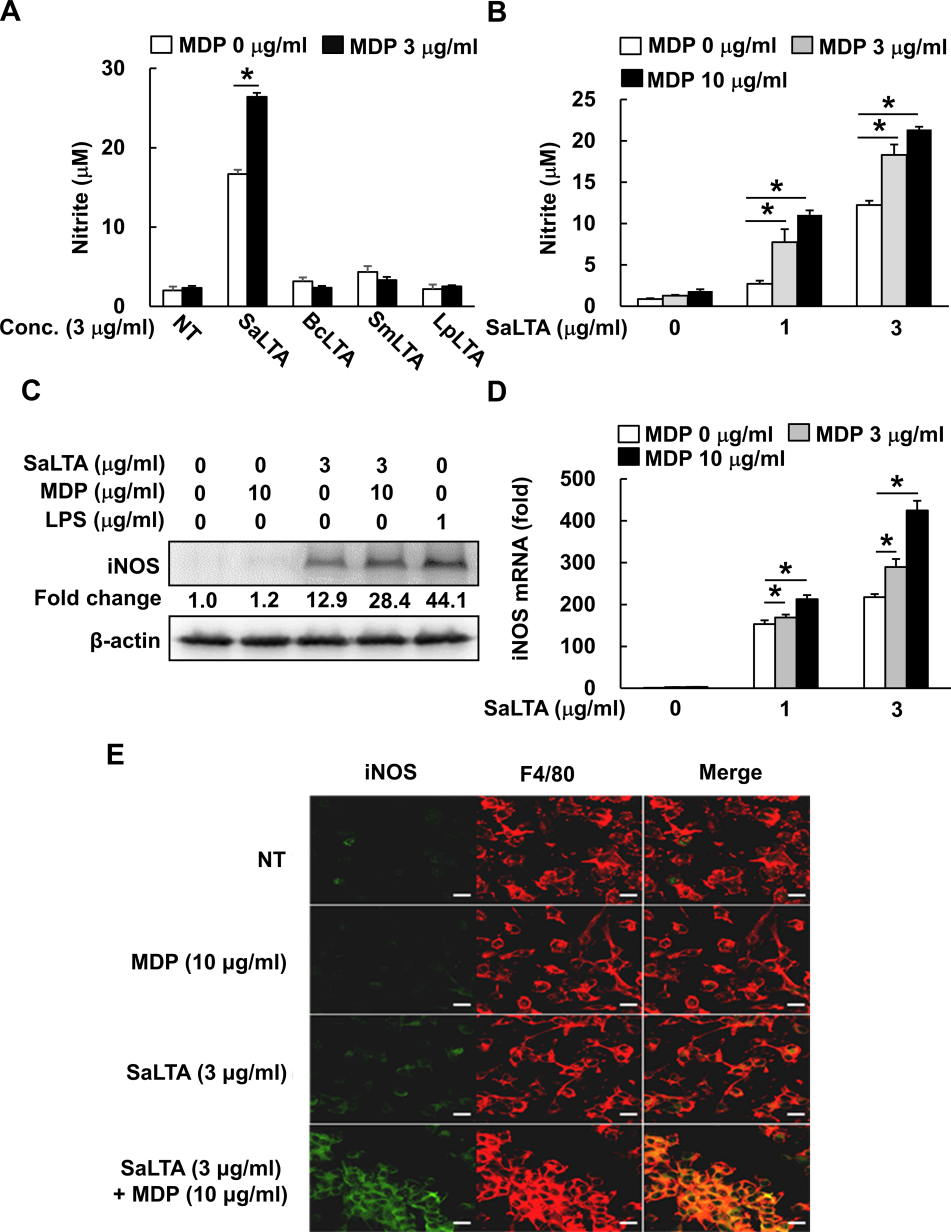
MDP enhances *S. aureus* LTA-induced iNOS and NO expression. **(A, B)** RAW 264.7 cells (200 μl of 5 × 10^5^ cells/ml) were treated with **(A)** MDP (3 µg/ml) and/or LTA (3 µg/ml) from *S. aureus* (SaLTA), *B. cereus* (BcLTA), *S. mutans* (SmLTA), or *L. plantarum* (LpLTA) for 24 h; or **(B)** various concentrations of SaLTA (0, 1, or 3 μg/ml) and/or MDP (0, 3, or 10 μg/ml) for 24 h. Supernatants were taken, and nitrite accumulation was measured as an indicator of NO production as described in the Materials and Methods section. NT, non-treatment group. **(C)** RAW 264.7 cells (5 ml of 5 × 10^5^ cells/ml) were treated with SaLTA (0 or 3 μg/ml) and/or MDP (0 or 10 μg/ml) for 12 h. The cell lysates were prepared using RIPA buffer and subjected to Western blot analysis using antibodies specific for iNOS or β-actin. The numbers given in figure represented the relative fold change in β-actin normalized iNOS expression of each group against that of non-treatment group set as 1 fold through densitometric analysis. **(D)** RAW 264.7 cells (3 ml of 5 × 10^5^ cells/ml) were treated with various doses of SaLTA (0, 1, or 3 μg/ml) and/or MDP (0, 3, or 10 μg/ml) for 12 h. Total RNA was prepared and used to synthesize cDNA. iNOS mRNA expression was determined by RT-qPCR. GAPDH was used as an internal control to normalize the iNOS mRNA expression. The data represent mean ± S.D. of three replicates for each group. Asterisk (*) indicates a significant difference compared to the control group at *P* < 0.05. **(E)** BMMs (500 μl of 1 × 10^4^ cells/ml) seeded onto glass coverslips were treated with SaLTA (0 or 3 μg/ml) and/or MDP (0 or 10 μg/ml) for 12 h. The cells were fixed with 4% paraformaldehyde and incubated with rabbit anti-mouse iNOS and rat anti-mouse F4/80 antibodies. Then, the coverslips were incubated with FITC-labeled goat anti-rabbit IgG and Cy3-labeled goat anti-rat IgG, and indirect fluorescence was detected by confocal microscopy. Green and red color shows iNOS and F4/80 expression, respectively. Scale bar given in each figure denotes 20 µm.

Since 10 µg/ml of MDP most potently enhanced SaLTA-induced NO production and iNOS expressions (**Figures 1B–D**), this concentration of MDP was selected for the further experiments, including flow cytometric analysis, luciferase reporter gene assay, Western blot analysis, and electrophoretic mobility shift assay. BMMs (500 μl of 1 × 10^4^ cells/ml) were seeded onto glass coverslips and treated with SaLTA (0 or 3 μg/ml) and/or MDP (0 or 10 μg/ml) for 12 h. After fixation using 4% paraformaldehyde, coverslips were washed with PBS and subsequently incubated with rabbit anti-mouse iNOS (Catalog No. ab3523, Abcam, Cambridge, *UK*) and rat anti-mouse F4/80 (Catalog No. SC-52664, Santa Cruz Biotechnology) antibodies for 1 h. The coverslips were incubated with FITC-labeled goat anti-rabbit IgG (Catalog No. 111-095-003, Jackson Immuno Research Laboratories, West Grove, PA, USA) and Cy3-labeled goat anti-rat IgG (Catalog No. 112-165-167, Jackson Immuno Research Laboratories) for 30 min. The coverslips were then subjected to confocal microscopy (LSM 510, Carl Zeiss, Oberkochen, Germany).

### Flow cytometry

RAW 264.7 cells (500 μl of 5 × 10^5^ cells/ml) were incubated with SaLTA (0 or 3 μg/ml) and/or MDP (0 or 10 μg/ml) for 24 h and stained with antibodies for TLR2 conjugated with PE, or CD14 conjugated with FITC for 30 min on ice (23). For intracellular NOD2 expression, the cells were initially fixed with 4% paraformaldehyde. The cells were permeabilized with 0.1% saponin and stained with antibody for NOD2 conjugated with PE for 30 min on ice. The cells were then subjected to flow cytometry (FACSCalibur, BD Biosciences).

### Luciferase reporter gene assay

RAW 264.7 cells (1 ml of 3 × 10^5^ cells/ml) were transfected with nuclear factor kappa B (NF-κB), or activator protein-1 (AP-1) luciferase reporter construct (Clontech, Palo Alto, CA, USA) using Lipofectamine plus reagent (Invitrogen, Grand Island, NY, USA) for 24 h. The cells were treated with SaLTA (3 μg/ml) and/or MDP (10 μg/ml) for an additional 24 h. At the end of the stimulation, the cells were lysed with Glo lysis buffer (Promega Corporation) and cytoplasmic extracts were collected by centrifugation at 13,000 *× g* and 4°C for 5 min. The luciferase activity in the cytoplasmic extracts was then measured using Bright-Glo luciferase assay system (Promega Corporation) and a luminometer (Victor 3, PerkinElmer, Waltham, MA, USA).

### Western blot analysis

RAW 264.7 cells (5 ml of 5 × 10^5^ cells/ml) were treated with SaLTA (0 or 3 μg/ml) and/or MDP (0 or 10 μg/ml) for 12 h for iNOS, and 2 or 4 h for STAT1. Then, the cell lysates were separated on 10% polyacrylamide gel and proteins on the gel were transferred to a PVDF membrane (Millipore, Bedford, MA, USA). After the incubation in 5% skim milk in Tris-buffered saline containing 0.1% Tween 20 for 1 h, the membrane was subsequently incubated with primary antibodies specific to iNOS, or phosphorylated or non-phosphorylated STAT1 at 4°C for overnight. Then, the membrane was incubated with HRP-conjugated anti-rabbit IgG (Sigma-Aldrich) for 1 h. The immuno-reactive bands on the membrane were visualized by ECL reagents (Amersham Biosciences, Princeton, NJ, USA) and an image analyzer (Fusion FX6.0, Vilber Lourmat, La Vallee, France). Data were analyzed with an image analysis program, ImageJ (NIH, Bethesda, MD, USA), and β-actin normalized protein expression levels were expressed as the relative fold change against non-treatment group set as 1 fold.

### Electrophoretic mobility shift assay

Nuclear extracts from RAW 264.7 cells (10 ml of 5 × 10^5^ cells/ml) stimulated with SaLTA (0 or 3 μg/ml) and/or MDP (0 or 10 μg/ml) were incubated with [γ-^32^P]-labeled deoxyoligonucleotide probes including the binding sites of NF-κB, or AP-1. Then, the mixtures were subjected to electrophoresis and autoradiography (23).

### Statistical analysis

All data were presented as mean values ± standard deviation (S.D.) from triplicate samples of each treatment group. Statistical significance was evaluated by Student’s *t*-test at *P* < 0.05.

## Results

### MDP potently enhances *S. aureus* LTA-induced iNOS and NO production

Based on the previous study, *S. aureus* PGN alone could not affect NO production in macrophages (13). On the other hand, according to our previous study, NO-inducing capacity of LTAs was different depending on their bacterial origin (20). Therefore, we initially purified LTAs from four Gram-positive bacteria, including three pathogenic bacteria (*S, aureus*, *S. mutans*, and *B. cereus*) and one beneficial bacterium (*L. plantarum*). Then, effects of those LTAs on NO production in macrophages in the presence or absence of MDP were examined. The result showed that *S. aureus* LTA (SaLTA) most potently increased NO production in RAW 264.7 cells compared to the other LTAs (**Figure 1A**). Although MDP alone did not affect NO production, MDP enhanced the SaLTA-induced NO production (**Figure 1A**). Next, we examined NO production on dose dependency of SaLTA and/or MDP. When the cells were stimulated with various concentrations of SaLTA (0-3 μg/ml), NO production was increased in a dose-dependent manner (**Figure 1B**). Furthermore, the SaLTA-induced NO production was potently enhanced in MDP dose-dependent manner (**Figure 1B**). Since iNOS is known as a key NO synthase in macrophage (24), iNOS expression in cells treated with SaLTA and/or MDP was examined. As shown in **Figures 1C, D**, iNOS expression was augmented by SaLTA, but not by MDP at both protein and mRNA levels. Like the NO production, MDP potently enhanced the SaLTA-induced iNOS expression (**Figures 1C, D**). To confirm the effects of MDP on SaLTA-induced iNOS expression, intracellular iNOS expression in BMMs treated with MDP and/or LTA was determined by immunofluorescence staining. In accordance with the results from the iNOS at protein and mRNA levels, MDP enhanced the SaLTA-induced iNOS expression (**Figure 1E**). These results demonstrated that MDP potentiates the inducibility of NO by SaLTA by enhancing the iNOS expression in macrophages.

### SaLTA-induced NO production enhanced by MDP is mediated through TLR2 and CD14/MyD88-, and NOD2-dependent pathway

**Figure 2:**
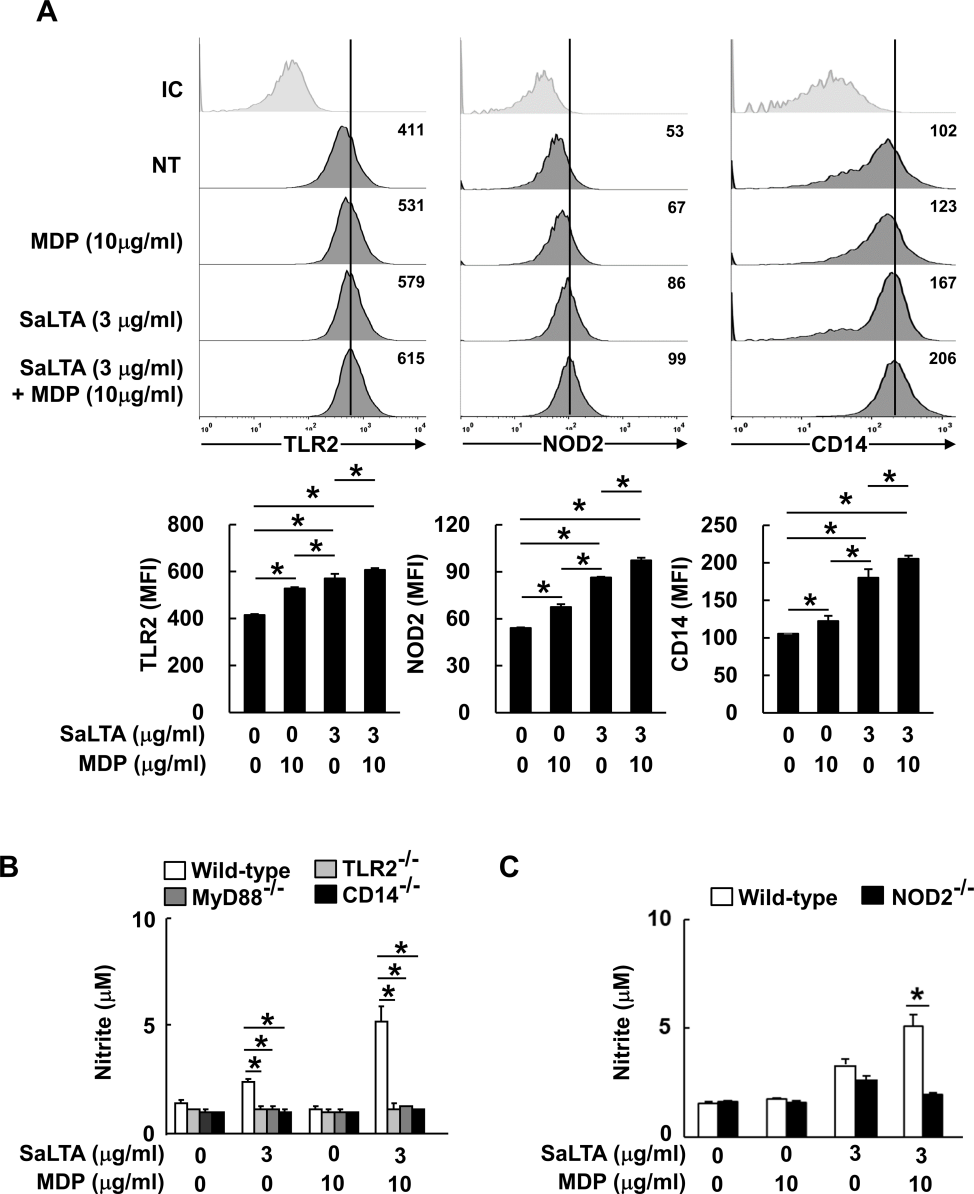
Enhanced SaLTA-induced NO production through MDP is mediated by TLR2 and CD14/MyD88-, and NOD2-dependent pathway. **(A)** RAW 264.7 cells (500 μl of 5 × 10^5^ cells/ml) were treated with SaLTA (0 or 3 μg/ml) and/or MDP (0 or 10 μg/ml) for 24 h. The cells were then stained with anti-mouse TLR2 conjugated with PE or CD14 conjugated with FITC antibodies. For intracellular NOD2, the cells were permeabilized and stained with anti-mouse NOD2 conjugated with PE. The stained cells were then subjected to flow cytometry. *Upper*, a representative TLR2, NOD2, and CD14 expression determined by flow cytometric analysis. Values given in each histogram indicate mean fluorescence intensity (MFI) of 10,000 events of live cells in the treatment group. *Lower*, the expression of TLR2, NOD2, and CD14 is presented as mean of MFI ± S.D. of three replicates for each group. Asterisk (*) indicates a significant difference between the indicated treatment groups at *P* < 0.05. NT, non-treatment group; IC, isotype control. **(B, C)** BMMs (200 μl of 5 × 10^5^ cells/ml) from wild-type, **(B)** TLR2-, MyD88- CD14- or **(C)** NOD2-deficient mice were treated with SaLTA (0 or 3 μg/ml) and/or MDP (0 or 10 μg/ml) for 24 h. Then, nitrite levels in the supernatants were measured. The data represent mean ± S.D. of three replicates for each group. * indicates a significant difference compared to the control group at *P* < 0.05.

LTA is known to be sensed by TLR2 together with its co-receptor CD14 (15), while MDP is sensed by NOD2 (16). Thus, we initially examined the expression of potential receptors involved in the sensing of SaLTA and MDP since the enhanced induction of NO by SaLTA and MDP may be due to the increased expression of those receptors. When basal expression levels of receptors, including TLR2, NOD2, and CD14, were examined, TLR2 and CD14 expressions were relatively higher than that of NOD2 (**Figure 2A**). While both SaLTA and MDP significantly increased the expression of all receptors examined, SaLTA more potently enhanced the expression of these receptors than MDP (**Figure 2A**). On the other hand, when the cells were treated with SaLTA and MDP together, all receptor expressions were augmented compared to those with MDP or LTA alone (**Figure 2A**). To further estimate the roles of these receptors in NO production in macrophages treated with SaLTA and MDP, we examined NO production in BMMs from TLR2-, CD14-, and NOD2-deficient mice (TLR2^-/-^, CD14^-/-^, and NOD2^-/-^) in the presence of SaLTA and MDP. The result showed that SaLTA-induced NO production was potently enhanced by MDP in BMMs from wild-type mice whilst it was not observed in BMMs from TLR2^-/-^, CD14^-/-^, and NOD2^-/-^ mice (**Figures 2B, C**). The recognition of LTA through TLR2/CD14 subsequently activated MyD88 and its down-stream molecules resulting in NO production (25). Thus, we examined the NO production in BMMs from MyD88-deficient mice (MyD88^-/-^) treated with SaLTA and MDP. As observed from the results from TLR2^-/-^, CD14^-/-^, and NOD2^-/-^ mice, SaLTA together with MDP did not induce significant NO production in BMMs from MyD88^-/-^ mice (**Figure 2B**). Collectively, these results suggest that TLR2-CD14/MyD88-, and NOD2-dependent pathways mediate the NO production by SaLTA and MDP in macrophages.

### Activation of MAP kinase is essential for the elevated SaLTA-induced NO production by MDP

**Figure 3:**
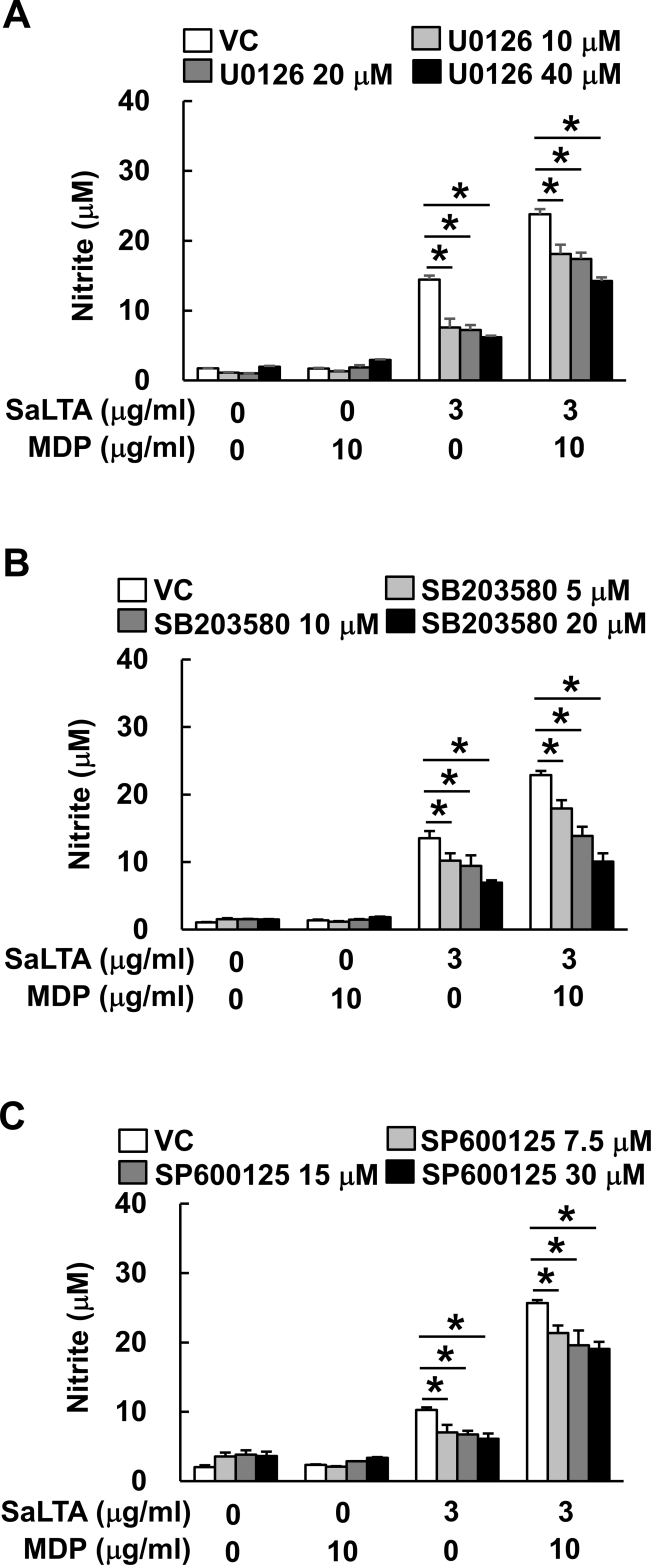
Activation of MAP kinase is essential for the elevated SaLTA-induced NO production by MDP. RAW 264.7 cells (200 μl of 5 × 10^5^ cells/ml) were pretreated with 0-40 μM of the indicated MAP kinase inhibitors, such as **(A)** U0126, **(B)** SB203580, or **(C)** SP600125 for ERK, p38, and JNK inhibition, respectively, for 1 h followed by treatment with SaLTA (0 or 3 μg/ml) and/or MDP (0 or 10 μg/ml) for 24 h. DMSO (0.1%) was used as a vehicle control (VC) for each MAP kinase inhibitor. After the treatment, nitrite levels in the supernatants were examined. The data represent mean ± S.D. of three replicates for each group. * indicates a significant difference compared to the control group at *P* < 0.05.

MyD88- and NOD2-dependent pathways commonly promote MAP kinase phosphorylation, subsequently causing the activation of transcription factors, such as AP-1 and STAT1 (15, 16). Moreover, it has been reported that activation of MAP kinases is important in inducing iNOS expression (26). To determine the involvement of MAP kinases in the enhanced induction of NO by SaLTA together with MDP, we evaluated the NO production in RAW 264.7 cells using MAP kinase-specific inhibitors, including U0126, SB203580, and SP600125 for extracellular signal-regulated kinase (ERK), p38, and c-Jun N-terminal kinase (JNK) inhibition, respectively. As shown in **Figures 3A–C**, all MAP kinase inhibitors attenuated the NO production by SaLTA alone or SaLTA with MDP. These results suggest that MAP kinases are essential for the enhanced NO production by SaLTA and MDP.

### NF-κB and AP-1 activations are responsible for the augmented SaLTA-induced NO production by MDP

**Figure 4:**
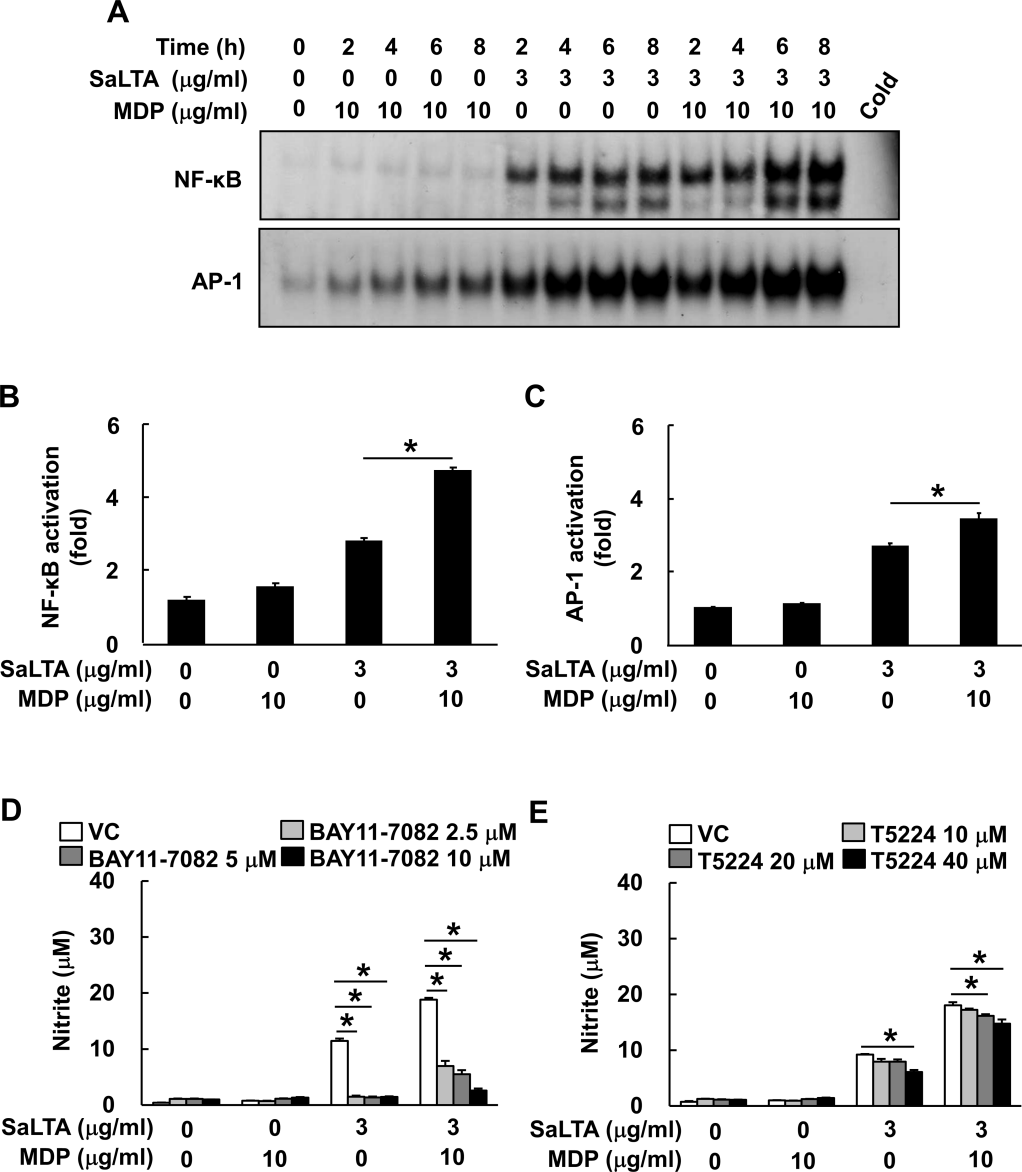
NF-κB and AP-1 activations are responsible for the augmented SaLTA-induced NO production by MDP. **(A)** Nuclear extracts were prepared from RAW 264.7 cells (10 ml of 5 × 10^5^ cells/ml) treated with SaLTA (0 or 3 μg/ml) and/or MDP (0 or 10 μg/ml) for 0, 2, 4, 6, or 8 h and incubated with ^32^P-labeled oligonucleotides containing NF-κB or AP-1 consensus sequences. One picomole of an unlabeled probe (Cold) was used in a competition assay to confirm specific binding. Reaction products were separated on a 4.8% polyacrylamide gel and visualized by autoradiography. **(B, C)** RAW 264.7 cells (1 ml of 3 × 10^5^ cells/ml) were transfected with **(B)** NF-κB or **(C)** AP-1 luciferase reporter construct for 24 h. The transfected cells were treated with SaLTA (3 μg/ml) and/or MDP (10 μg/ml) for an additional 24 h. After the treatment, cytosolic extracts were subjected to the luciferase activity assay as described in the Materials and Methods section. **(D, E)** RAW 264.7 cells (200 μl of 5 × 10^5^ cells/ml) were pretreated with 0-40 μM of **(D)** BAY11-7082, or **(E)** T5224 for 1 h followed by treatment with SaLTA (0 or 3 μg/ml) and/or MDP (0 or 10 μg/ml) for 24 h. DMSO (0.1%) was used as a vehicle control (VC) for each inhibitor. Then, nitrite levels in the supernatants were measured. The data represent mean ± S.D. of three replicates for each group. * indicates a significant difference compared to the control group at *P* < 0.05.

Among various transcription factors, NF-κB (27) and AP-1 activations by MAP kinases (26) are known to be involved in iNOS expression. As shown in **Figure 4A**, SaLTA and MDP enhanced more DNA-binding activity of both NF-κB and AP-1 transcription factors than SaLTA did alone at 6 and 8 h after the treatment. In accordance with the results from EMSA, the results of luciferase reporter gene assay showed augmented NF-κB and AP-1 activities by SaLTA together with MDP compared to SaLTA alone (**Figures 4B, C**). Next, we evaluated the role of these transcription factors on the synergistic NO production using BAY11-7082, which is a broad-spectrum inhibitor for various intracellular signaling mediators, including NF-κB and AP-1 (28), and T5224, a specific inhibitor for AP-1. The SaLTA/MDP-induced NO production was suppressed by each of BAY11-7082 and T5224 (**Figures 4D, E**). Thus, these results demonstrated that NF-κB and AP-1 activations in macrophages treated with SaLTA and MDP are responsible for the induction of NO.

### STAT1 activation by PAFR contributes to the induction of NO by SaLTA and MDP

**Figure 5:**
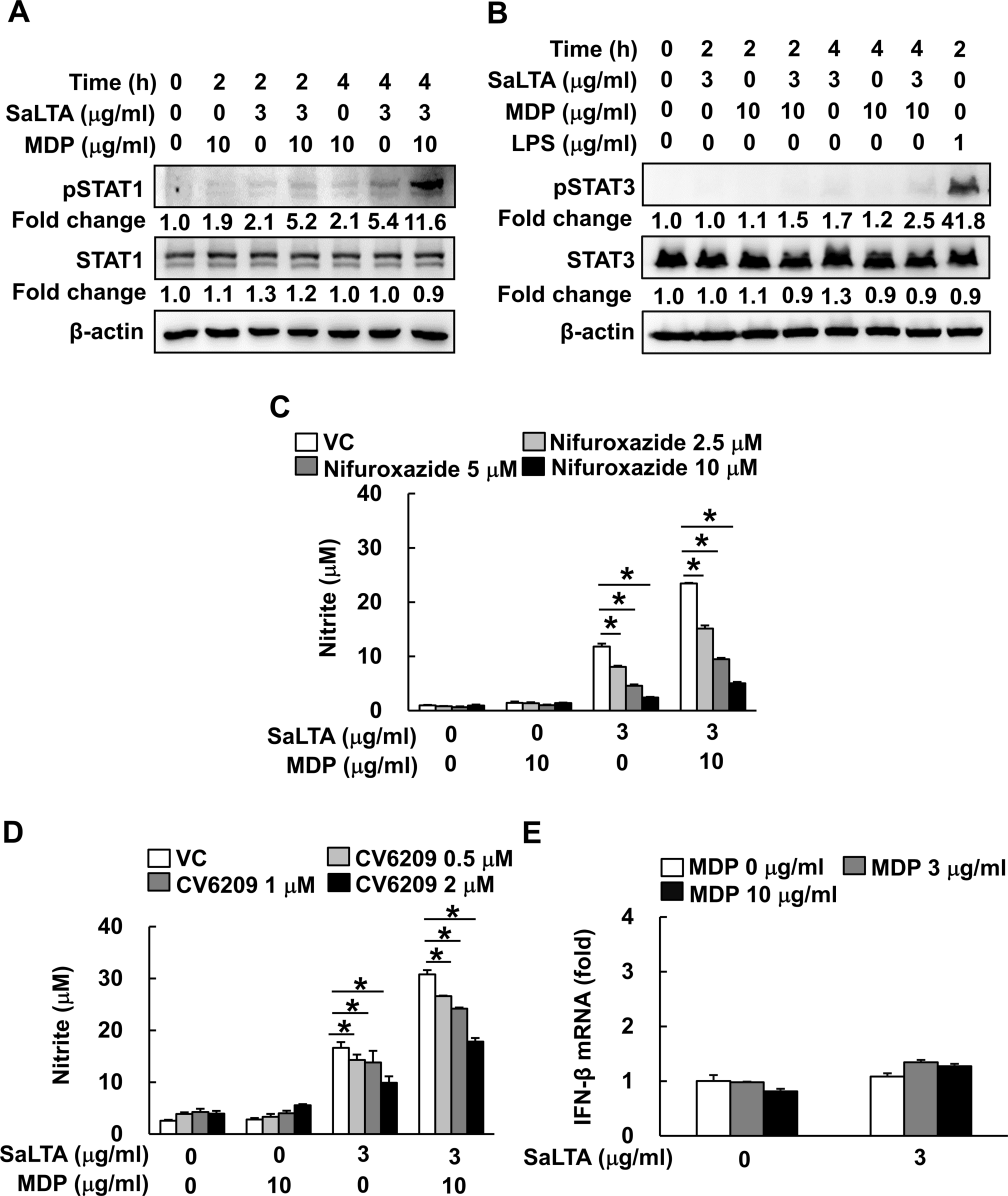
STAT1 activation by PAFR contributes to the induction of NO by SaLTA and MDP. **(A, B)** The cell lysates were prepared from RAW 264.7 cells (5 ml of 5 × 10^5^ cells/ml) treated with SaLTA (0 or 3 μg/ml) and/or MDP (0 or 10 μg/ml) for 0, 2, or 4 h and subjected to Western blot analysis using antibodies specific for β-actin, non-phosphorylated or phosphorylated forms of **(A)** STAT1 or **(B)** STAT3. The numbers given in figure represented the relative fold change in β-actin normalized non-phosphorylated and phosphorylated **(A)** STAT1 or **(B)** STAT3 expression of each group against that of that of non-treatment group set as 1 fold through densitometric analysis. **(C, D)** RAW 264.7 cells (200 μl of 5 × 10^5^ cells/ml) were pretreated with various concentrations of inhibitors, such as **(C)** nifuroxazide or **(D)** CV6209, for STAT1 and STAT3, and PAFR inhibition, respectively, for 1 h followed by treatment with SaLTA (0 or 3 μg/ml) and/or MDP (0 or 10 μg/ml) for 24 h. Then, nitrite levels in the cultured media were measured. DMSO (0.1%) was used as a vehicle control (VC). **(E)** RAW 264.7 cells (3 ml of 5 × 10^5^ cells/ml) were treated with SaLTA (0 or 3 μg/ml) and/or MDP (0, 3, or 10 μg/ml) for 12 h. Total RNA was prepared using TRIzol reagent and was used to synthesize cDNA. IFN-β mRNA expression was determined by RT-qPCR. GAPDH was used as an internal control to normalize the IFN-β mRNA expression. The data represent mean ± S.D. of three replicates for each group. * indicates a significant difference compared to the control group at *P* < 0.05.

Furthermore, STAT1 is also a major transcription factor responsible for the regulation of iNOS via PAFR-dependent pathway (29). Thus, we initially examined the effect of SaLTA and/or MDP on phosphorylation of STAT1 in RAW 264.7 cells. Activation of STAT1 was observed in the cells treated with SaLTA, but not with MDP (**Figure 5A**). Interestingly, SaLTA together with MDP more potently induced activation of STAT1 than SaLTA did alone. On the other hand, it has been recently reported that STAT3 can act as a major transcription factor responsible for the regulation of IL-6 and IL-11 production via PAFR-dependent pathway in tumor cells and cancer-associated fibroblasts (30), suggesting the potential role of STAT3 in the enhanced SaLTA-induced NO production by MDP via PAFR dependent pathway. For this reason, we also examined the effect of SaLTA and/or MDP on phosphorylation of STAT3 in RAW 264.7 cells and found that STAT3 phosphorylation was rarely induced by SaLTA and/or MDP (**Figure 5B**). Next, we examined the role of STAT1 in the SaLTA and MDP-induced NO production using STAT1 and 3 inhibitor nifuroxazide. The NO production in the cells treated with SaLTA and MDP was inhibited by nifuroxazide in a dose-dependent manner (**Figure 5C**). Furthermore, to evaluate the role of PAFR in the NO production, we examined the NO production in the cells treated with SaLTA and MDP in the presence of PAFR inhibitor CV6209. As shown in **Figure 5D**, NO production by SaLTA alone or SaLTA together with MDP was suppressed by the inhibitor in a dose-dependent manner. Since STAT1 phosphorylation is also mediated by IFN-β via its hetero-dimer receptors, IFNAR1 and IFNAR2 (31), we tested whether SaLTA and MDP induce IFN-β mRNA expression in RAW 264.7 cells. However, IFN-β mRNA expression was not affected by SaLTA and/or MDP (**Figure 5E**). These results implied that the NO production is also mediated by enhanced STAT1 activity via PAFR induced by SaLTA together with MDP.

## Discussion

*S. aureus*, especially methicillin-resistant *S. aureus*, is reported as a major pathogen causing sepsis, and its pathogenicity is mediated by its virulence factors (3). Among the diverse cell wall constituents, LTA and PGN are regarded as major virulence factors of *S. aureus* based on their immuno-stimulating capacities (14, 16). Thus, investigating their interaction on NO production, a key molecule closely related to the initiation and progression of sepsis, might contribute to better understanding the pathogenesis of sepsis caused by *S. aureus* infection. Here, we found that MDP potently enhanced SaLTA-induced NO production as well as iNOS expression. Furthermore, we characterized the underlying mechanisms involved in the enhanced NO production by SaLTA and MDP. NO production in macrophages treated with SaLTA and MDP is mediated by TLR2/NOD2/PAFR, MAP kinases, and NF-κB/AP-1/STAT1 transcription factors.

Our results showed that MDP potentiates SaLTA-induced NO production in macrophages through the unique signaling cascade comprising TLR2-, NOD2- and PAFR-dependent pathways. Such cooperative effects of MDP on SaLTA-induced inflammatory responses have also been previously reported in diverse cells or tissues. Similar to our observation, MDP potentiated SaLTA-induced NO production in rat microglia cells (32). In addition, MDP synergistically enhanced SaLTA-induced IL-8 expression in human monocytes (33) and neutrophil recruitment to mammary glands through chemokine overproduction (34). On the other hand, although previous studies reported that LTA together with PGN from *S. aureus* induced sepsis and multi-organ dysfunction in rodent models (13, 17), the underlying mechanisms are not fully understood. In our previous study, we reported that MDP potentiated SaLTA-induced cyclooxygenase-2 expression in macrophages, which promotes prostaglandin E2 production subsequently leading to cause of detrimental effects such as vasodilation and increased vascular permeability during sepsis (35). Collectively, our current and previous studies provided potential mechanisms responsible for the inflammatory responses caused by *S. aureus* infection leading to sepsis via clarifying the cooperative effect of LTA and MDP on expression of various inflammatory molecules.

In the current study, together with TLR2/NOD2-dependent pathways, PAFR-dependent pathway seems to be also critical in the enhanced SaLTA-induced NO production by MDP. Based on previous studies, PAFR activation can be involved in NO production of macrophages and epithelial cells (36, 37). In addition, we previously reported that PAFR signaling pathway contributed to the staphylococcal or pneumococcal LTA-induced NO production (29). Furthermore, similar to the current study, *Bacillus anthracis* capsule-induced NO production was triggered by both TLR2- and PAFR-dependent signaling pathways in macrophages (38). Nevertheless, it is still questionable on how MDP potentiated the SaLTA-induced PAFR-dependent pathway. Since it has been reported that only phosphorylcholine-decorated cell wall components, such as peptidoglycan-teichoic acid complex, could bind to PAFR and get engulfed into endothelial and epithelial cells (39), MDP may not directly bind to PAFR. In our previous study, LTA, which is structurally similar to teichoic acid, resembles bioactive component of PAF, and it not only augments PAFR expression in BMM through TLR2 but also promotes production of PAF (21). We speculated that SaLTA-induced PAF might promote the PAFR-mediated macropinocytosis of macrophages, subsequently leading to MDP engulfment thereby enhancing the SaLTA-induced NO production. According to previous studies, the interaction between PAF and PAFR induces phosphatase activity of protein tyrosine phosphatase non-receptor type 2 (PTPN2) production resulting in the activation of PI3K/Akt pathways (40). Moreover, since the activated enzymatic activity of PI3K contributes to formation of extended lamellipodia and ruffles required for macropinocytosis (41), the activated PAFR pathway by SaLTA-induced PAF might enhance engulfment of MDP via the macropinocytosis. Then, the engulfed MDP can be trafficked to endosome and egress to cytosol by two endo-lysosomal peptide transporters (42). Finally, MDP released from endosome would enhance activity of iNOS through NOD2 activation (43) and subsequently potentiate SaLTA-induced NO production. To clarify exact mechanisms responsible for up-regulated PAFR-dependent pathway by SaLTA and MDP, such possibilities should be verified through further studies.

Among the various LTAs tested in this study, SaLTA was the most potent to induce NO production. The differential NO inducibilities of various LTAs may be caused by their structural diversity. LTA can be classified into two major structural classes, including glycerophosphate- and ribitolphosphate-types (44). LTAs tested in the current study belong to glycerophosphate-type LTA and are composed of repetitious glycerophosphate chains linked to a lipid moiety (44). The lipid moiety of LTA is important in determining its immunostimulatory capacity. In fact, LpLTAs, possessing unsaturated fatty acids in their lipid moiety, have relatively weaker immunostimulatory capacity than LTAs with saturated fatty acids, such as SaLTA (45). This difference may be resulted from LTAs having saturated fatty acids easily interact with cellular membrane lipid rafts, which are composed of saturated fatty acids (46), subsequently leading to more activation of TLR2-dependent signaling (47). On the other hand, LTAs with less glycerophosphate chain with less D-alanine content in its glycerophosphate chain have low immunostimulatory potency (19).

In the current study, although NOD2 is expressed in cytosolic part of BMMs, MDP did not affect NO production. Similar to our results, a previous study also demonstrated that MDP alone did not affect NO production in BMMs without IFN- γ pretreatment (48). On the other hand, other previous studies reported that the MDP can induce NO production in the thioglycollate-elicited peritoneal macrophages (TG-PMs) (49). Since BMMs express relatively higher levels of pattern recognition receptors (PRRs), including TLR2 needed for SaLTA recognition, compared to TG-PMs (50), BMMs were used rather than other tissue-derived macrophages in the current study. In addition, despite the similarities between TG-PMs and BMMs in sizes and granularities, BMMs are known to exhibit more responsiveness in phagocytic capacity and cytokine expression than TG-PMs (50). Furthermore, peritoneal lavage contains various cell types other than macrophages, such as neutrophils and eosinophils (50, 52), that might be involved in MDP-induced NO production. Indeed, eosinophils in the PM preparations can hinder accurate interpretation of results from *in vitro* study using PMs (53). For these reasons, we considered BMMs to be a more suitable model than TG-PMs for investigating macrophage plasticity and immune responses during bacterial infection.

During microbial infection, excessive NO production in the systemic circulation causes a profound vasodilatation followed by a systemic hypotension and microvascular hyperpermeability, and if severe, leading to the death of patient (51). Moreover, since elevated NO production causes severe sepsis, NOSs, especially iNOS, are considered as target molecules for the development of sepsis therapeutics (11). During the past three decades, bacteria causing sepsis have been dramatically shifted from Gram-negative to Gram-positive pathogens, especially *S. aureus* (3). However, mechanism studies to identify key etiological agents inducing NO expression have mainly focused on LPS from Gram-negative pathogen rather than on virulence factors of *S. aureus*. Here, we found that MDP potently enhanced SaLTA-induced NO production as well as the underlying intracellular mechanisms related to the NO production. Collectively, our current study provides potential clues on the understanding of pathology of sepsis by *S. aureus*, Gram-positive pathogen, and contributes to developing potential therapeutics for sepsis under Gram-positive bacterial infection.

## Data Availability

The original contributions presented in the study are included in the article/supplementary material. Further inquiries can be directed to the corresponding author.
